# Thermo-Optic Response and Optical Bistablility of Integrated High-Index Doped Silica Ring Resonators

**DOI:** 10.3390/s23249767

**Published:** 2023-12-11

**Authors:** Junkai Hu, Jiayang Wu, Di Jin, Sai Tak Chu, Brent E. Little, Duan Huang, Roberto Morandotti, David J. Moss

**Affiliations:** 1 Optical Sciences Center, Swinburne University of Technology, Hawthorn, VIC 3122, Australia; junkaihu@swin.edu.au (J.H.);; 2 School of Automation, Central South University, Changsha 410083, China; 3 Department of Physics, City University of Hong Kong, Hong Kong SAR 999077, China; 4 QXP Technology Inc., Xi’an 710119, China; brent.little@qxptech.com; 5 School of Computer Science and Engineering, Central South University, Changsha 410083, China; 6 EMT (Énergie Matériaux Télécommunications Research Centre), INRS (Institut National de la Recherche Scientifique), Varennes, QC J3X 1S2, Canada; morandotti@emt.inrs.ca

**Keywords:** integrated optics, thermo-optic effects, microring resonator, optical bistability

## Abstract

The engineering of thermo-optic effects has found broad applications in integrated photonic devices, facilitating efficient light manipulation to achieve various functionalities. Here, we perform both an experimental characterization and a theoretical analysis of these effects in integrated microring resonators made from high-index doped silica, which have had many applications in integrated photonics and nonlinear optics. By fitting the experimental results with theory, we obtain fundamental parameters that characterize their thermo-optic performance, including the thermo-optic coefficient, the efficiency of the optically induced thermo-optic process, and the thermal conductivity. The characteristics of these parameters are compared to those of other materials commonly used for integrated photonic platforms, such as silicon, silicon nitride, and silica. These results offer a comprehensive insight into the thermo-optic properties of doped silica-based devices. Understanding these properties is essential for efficiently controlling and engineering them in many practical applications.

## 1. Introduction

The heat management and control of optical devices is of fundamental importance to their practical applications [1,2]. For integrated photonic devices with a compact footprint and tight mode confinement and particularly for materials that do not exhibit second-order optical nonlinearities such as the Pockels effect [3], the importance of precisely engineering their thermo-optic effects is even more pronounced [4,5]. Over the past decade, with the rapid advancement in integrated photonics, extensive research has been dedicated to investigating and harnessing thermo-optic effects to manipulate light in integrated photonic devices, particularly those based on centrosymmetric materials [4,6]. This has enabled the realization of a variety of functionalities such as mode locking [7,8], optical switches [9,10], logic gates [11], power limiters [11,12], optical memories [11,13,14], and sensors [15,16].

As an important complementary metal–oxide–semiconductor (CMOS)-compatible integrated platform, high-index doped silica has been extensively utilized in diverse linear and nonlinear integrated photonic devices for a range of applications [17,18]. High-index doped silica possesses a host of attractive optical properties, such as low linear optical absorption over a broad band, a reasonably strong Kerr nonlinearity (about five times that of silica), and negligible nonlinear optical absorption [19,20]. The combination of these properties and its strong compatibility with the globally established CMOS infrastructure contributes to the exceptional performance and versatility of doped silica devices in various applications within the field of integrated photonics.

Despite the proven success of high-index doped silica devices in many optical applications based on their extremely low linear loss and excellent nonlinear optical properties, investigation of the thermo-optic effects in these devices has not been as extensive as in integrated photonic devices made from other materials such as silicon and silicon nitride [14,21,22]. There remains a need for the exploration and understanding of the thermo-optic properties of high-index doped silica devices to fully leverage their potential in integrated photonics. In this paper, we address this issue by providing a comprehensive experimental characterization and theoretical analysis of these effects in high-index doped silica integrated devices. By fitting the experimental results with theory, we obtain fundamental parameters that characterize the thermo-optic properties of high-index doped silica devices, including the thermo-optic coefficient, the efficiency of the optically induced thermo-optic process, and the thermal conductivity. We also provide a comparison of these parameters with those of other materials used for CMOS-compatible integrated photonic platforms, such as silicon, silicon nitride, and silica. These findings provide a comprehensive understanding of the thermo-optic properties of high-index doped silica devices, which is important for effectively controlling and engineering these devices in many applications.

## 2. Device Fabrication and Characterization

**Figure 1:**
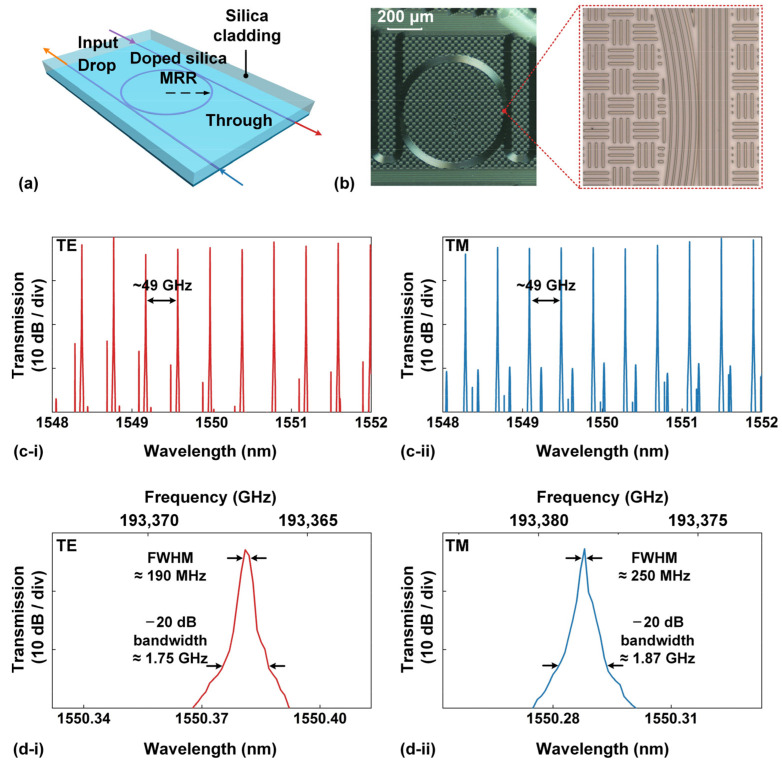
(**a**,**b**) Schematic and microscopic image of an add-drop microring resonator (MRR) made from high-index doped silica, respectively. Inset shows zoomed-in view of the coupling region. (**c**) Measured transmission spectra of the doped silica MRR for (**i**) TE and (**ii**) TM polarizations. (**d**) Zoomed-in views of single (**i**) TE- and (**ii**) TM-polarized resonances at ~1550.381 nm and ~1550.288 nm, respectively.

Figure 1a shows a schematic of an add-drop MRR made from high-index doped silica. A microscope image of the fabricated device is shown in Figure 1b, and the inset shows a zoomed-in view of the coupling region. The fabrication process has been discussed previously [17,19,20,23,24]. First, a lower cladding layer of SiO_2_ was deposited via low-temperature plasma-enhanced chemical vapor deposition (PECVD). The PECVD pressure and temperature were designed to be back-end CMOS compatible, at a pressure of ~1 atm and temperature below 400 °C, respectively. The high-index doped silica film core with a thickness of ~2 μm was deposited using the same process, and the refractive index contrast could be adjusted from 0% to over 20%. In our fabrication, the waveguides in the MRR had a core index of ~1.66 and an index contrast of 14.4%. Next, waveguides with exceptionally low sidewall roughness were formed by employing deep-ultraviolet photolithography techniques and reactive ion etching. Finally, a silica layer with a refractive index of ~1.45 was deposited via PECVD as the upper cladding. The waveguide cross section of both the MRR and the two coupling bus waveguides was ~3 µm × ~2 µm. The MRR had a radius of ~592.1 µm, which corresponded to a free spectral range (FSR) of ~0.4 nm (i.e., ~49 GHz). Note that, although there are a number of concentric rings in Figure 1b, only the central ring was coupled with the through/drop bus waveguides to form an MRR with a radius of ~592.1 µm, and the rest were simply used to enable easy identification by eye. In our previous work, Ref. [20], a similar MRR layout was used except that the chip was planarized to remove the upper silica cladding. The input and output ports of the MRR were connected to specially designed mode converters that were packaged with fiber pigtails. The fiber-to-chip coupling loss was ~1.5 dB/facet, with this low value enabled through the use of on-chip mode converters to the pigtailed fibers.

Figure 1c shows the measured transmission spectra of a fabricated high-index doped silica MRR for both transverse magnetic (TE) and transverse electric (TM) polarizations. The wavelength of a tunable continuous-wave (CW) laser was scanned at a constant input power of ~0 dBm to measure the transmission spectra, and a polarization controller (PC) was employed to adjust the input polarization. The input power here and in our following analysis refers to the power coupled into the device (i.e., the on-chip power), with the fiber-to-chip coupling loss being subtracted from the laser’s output power. The free spectral range (FSR) of the TE- and TM-polarized transmission spectra was ~0.4 nm, which corresponded to ~49 GHz. By tuning the PC, the maximum polarization extinction ratios for the TE- and TM-polarized resonances were >30 dB.

Figure 1d shows zoomed-in views of single TE- and TM-polarized resonances at ~1550.381 nm and ~1550.288 nm. There was no significant asymmetry in the measured resonance spectral line shape, indicating that the thermal effect at the input power of ~0 dBm was negligible. The full widths at half maximum (FWHMs) of the TE- and TM-polarized resonances were ~0.0015 nm (~190 MHz) and ~0.0020 nm (~250 MHz), respectively, which corresponded to Q factors of ~1.0 × 10^6^ and ~7.8 × 10^5^, respectively. In addition, the −20 dB bandwidths of the TE- and TM-polarized resonances were ~1.75 GHz and ~1.87 GHz, respectively. By using the scattering matrix method [25] to fit the measured spectra in Figure 1d, we obtained the device parameters for the doped silica MRR that were used for the analysis in the subsequent sections. These parameters, together with the specific material and waveguide parameters, are summarized in Table 1.

## 3. Thermo-Optic Coefficient

The thermo-optic coefficient of a material is a fundamental parameter that indicates how its refractive index changes with environmental temperature, which plays an important role in the design and engineering of relevant devices [26], for examples, for sensing applications [27,28]. In this section, we characterize the thermo-optic coefficient of high-index doped silica by measuring the transmission spectra of the doped silica MRR with varying chip temperatures.

**Figure 2:**
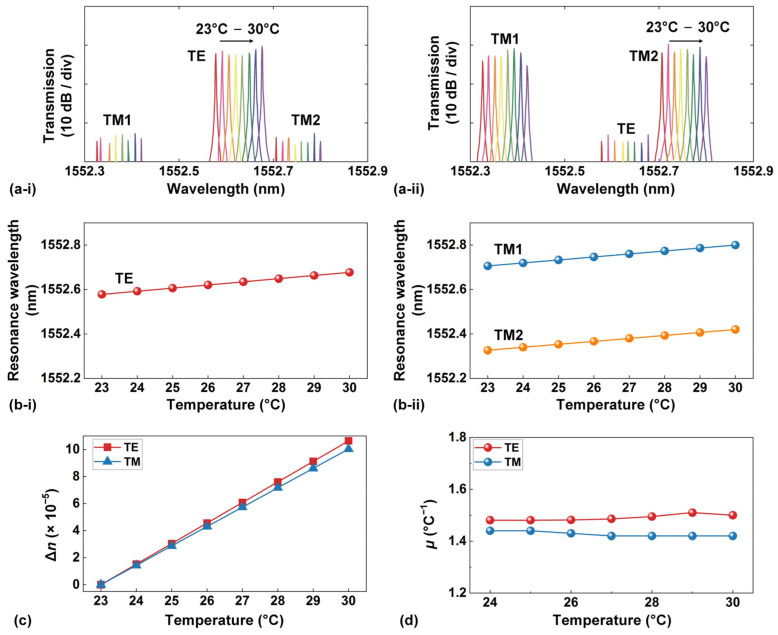
(**a**) Measured (**i**) TE- and (**ii**) TM-polarized transmission spectra of high-index doped silica MRR when the chip temperature changes from 23 °C to 30 °C, respectively. The results presented depict a resonance with TE polarization positioned between two resonances with TM polarization (TM1 and TM2). (**b**) Resonance wavelength shifts versus chip temperature for (**i**) TE and (**ii**) TM polarizations extracted from (**a**). (**c**) Changes in waveguide effective refractive indices versus chip temperature extracted from (**b**). (**d**) Thermo-optic coefficient μ versus chip temperature extracted from (**c**).

When there are changes in environmental temperature, the thermo-optic effect causes changes in the effective refractive index of the high-index doped silica waveguides. Consequently, this leads to a shift in the resonance wavelengths of the doped silica MRR. Figure 2a shows the TE- and TM-polarized transmission spectra of the doped silica MRR when the chip temperature changed from 23 °C to 30 °C, respectively. We measured the shifts of three resonances, including a TE-polarized resonance and two TM-polarized resonances (TM1 and TM2). Especially, the TE-polarized resonance was located between the two TM-polarized resonances. To adjust the temperature of the integrated chip mounted on a stage, a temperature controller was employed. The input power of the scanned CW laser was maintained as ~0 dBm (i.e., the same as that in Figure 1) in order to mitigate noticeable thermal effects. It is important to highlight that, despite the changes in environmental temperature, no significant asymmetry was observed in the measured resonance spectral line shape. This observation indicates that changes in environmental temperature induced by the temperature controller have a minimal impact on the asymmetry of the resonance spectral line shape and do not induce significant optical bistability, which will be discussed in the next section [29].

Figure 2b shows the resonance wavelength shifts versus the chip temperature, which were extracted from the results in Figure 2a. The TE-polarized resonance redshifted at a rate of ~14.2 pm/°C, whereas the two TM-polarized resonances exhibited a redshift rate of ~13.4 pm/°C. Figure 2c depicts the changes in the waveguide effective refractive indices versus the chip temperature. These results were calculated using the measured results in Figure 2b, along with the relationship between the resonance wavelengths and the waveguide effective refractive index as follows [30]:

(1) $$n_{eff}\times 2\pi /\lambda_{m}\times L=\;m\times 2\pi$$

where *n_eff_* is the effective refractive index of the high-index doped silica waveguide, *L* is the circumference of the doped silica MRR, and *m* represents the *m*th resonance, with *λ_m_* denoting the corresponding resonance wavelength.

In Figure 2c, the TE mode displays a change in the effective refractive index at a rate of ~1.52 × 10^−5^/°C, while the TM mode changes at a rate of ~1.43 × 10^−5^/°C. The difference in these rates can be attributed to the asymmetric cross section of the doped silica waveguide. Based on these results, we further extracted the thermo-optic coefficient of the high-index doped silica material at various chip temperatures by using Lumerical FDTD commercial mode solving software (Lumerical 2014a). The results are presented in Figure 2d. In our simulation, the thermo-optic coefficient of silica was assumed to be ~1.09 × 10^−5^/°C [31]. Given the low thermal expansion coefficient of silica (~0.5 × 10^−6^/K [32]) as well as the relatively large waveguide dimensions for our doped silica waveguides (~3 µm × ~2 µm), we did not account for the thermal expansion of the high-index doped silica waveguides. The thermo-optic coefficients of doped silica in Figure 2d do not show significant temperature dependence. We also note that the average values of the thermo-optic coefficients of doped silica derived from the TE and TM modes exhibited remarkable similarity, at ~1.49 × 10^−5^/°C and ~1.44 × 10^−5^/°C, respectively. This close resemblance between the coefficients reflects that high-index doped silica does not exhibit significant anisotropy in terms of its thermo-optic coefficient.

## 4. Optically Induced Thermo-Optic Response

When a material is illuminated with intense light, optical absorption leads to heat generation that raises the local temperature. This in turn modifies the material’s refractive index, thereby influencing the propagation of light through the material. In this optically induced thermo-optic process, the change in the material’s refractive index *n* due to the temperature variation induced by the optical field can be modeled as follows [21,33]:

(2) $$n=n_{0}+{\overset{-}{n}}_{2}\times I$$

where *n*_0_ is the material’s refractive index when not exposed to light, and ${\overset{-}{n}}_{2}$ × *I* is the refractive index change due to the optically induced temperature change, with *I* denoting the light intensity and ${\overset{-}{n}}_{2}$ denoting the coefficient that characterizes the efficiency for this process. In this section, we characterize the ${\overset{-}{n}}_{2}$ of high-index doped silica by measuring the transmission spectra of the doped silica MRR at various input powers. It is worth noting that Equation (2) is the same as that used for modeling the nonlinear Kerr optical effect [34,35]. For the optically induced refractive index change, in addition to the optically induced thermo-optic effect, there will also be a presence of the Kerr optical effect. Despite having the same mathematical modeling as shown in Equation (2), these two effects are associated with different physical processes that exhibit distinct characteristics. For example, compared to the Kerr optical effect that has an ultrafast time response on the order of 10^−15^ s [36], the time response for the optically induced thermo-optic effect is much slower, typically on the order of 10^−6^–10^−3^ s [37,38,39,40,41].

**Figure 3:**
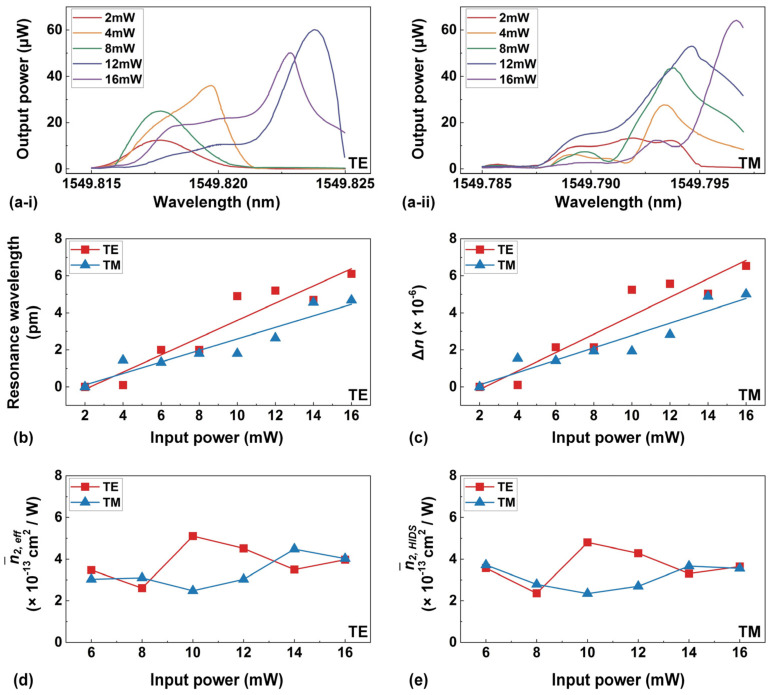
(**a**) Measured transmission spectra of high-index doped silica MRR at varying input powers for (**i**) TE and (**ii**) TM modes. (**b**) Measured (data points) and fitted (solid curves) resonance wavelength shifts versus input power. (**c**) Waveguide effective refractive index changes versus input power extracted from (**b**). (**d**) ${\overset{-}{n}}_{2,\;eff}$ versus input power extracted from (**c**). (**e**) ${\overset{-}{n}}_{2,\;\mathrm{doped}\;\mathrm{silica}}$ versus input power extracted from (**d**).

When the wavelength of incident light is on resonance with the MRR, the incident light power converts into heat more efficiently, being enhanced significantly by the ring resonance, leading to an efficient change in the effective refractive index of the high-index doped silica waveguides caused by the thermo-optic effect. This refractive index change also results in a shift in the resonance wavelengths of the doped silica MRR. Figure 3(a-i,a-ii) show the measured transmission spectra of the doped silica MRR at different input powers for TE and TM polarizations, respectively. As the input power increased, a redshift in the resonance wavelengths was observed, accompanied by increasingly asymmetric resonance spectra. The spectra also exhibited a steepened transition edge, indicating the presence of the optical bistability [42,43].

Depending on the dominating nonlinear mechanism, the resonance wavelengths can experience either a blue or redshift. In previous work on bistability in silicon MRRs at room temperature, it was observed that the resonance wavelengths initially exhibited a blueshift and subsequently transitioned to a redshift as the input power increased [44]. This is because the free-carrier dispersion (FCD) that results in a decreased refractive index of silicon dominates at low powers, whereas the thermo-optic effect that leads to an increased refractive index dominates at high powers [44]. Here, we only observed a redshift in the resonance wavelengths, mainly due to the dominating thermo-optic effect and negligible FCD for the high-index doped silica MRR [20] and the fact that the magnitude of the all-optical Kerr component of the index change tends to be much smaller for typical CW powers.

Figure 3b shows the shifts of the resonance wavelength versus the input power. For both the TE and TM polarizations, the positive Δ*λ* (which indicates a redshift) exhibits a nearly linear relationship with the input power. By linearly fitting the measured results, we obtained the rates for the resonance wavelength shift, which were ~0.4655 pm/mW and ~0.3144 pm/mW for the TE and TM polarizations, respectively.

Figure 3c shows the changes in the waveguide effective refractive indices versus the input power for both TE and TM polarizations. These results were calculated based on Equation (1), using the measured results in Figure 3b. As the input power increased from ~2 mW to ~16 mW, the effective refractive indices of the TE and TM modes displayed changes of ~6.533 × 10^−6^ and ~5.012 × 10^−6^, respectively. These changes correspond to average rates of ~4.985 × 10^−7^/mW and ~3.335 × 10^−7^ /mW, respectively.

In Equation (2), ${\overset{-}{n}}_{2}$ can also be an effective response for the MRR, in that it is device geometry dependent, including the Q factor, coupling strength, etc. Figure 3d shows the MRR’s effective ${\overset{-}{n}}_{2}$, denoted as ${\overset{-}{n}}_{2,\;eff}$, versus the input power for both TE and TM polarizations, which were extracted from the results in Figure 3c. The ${\overset{-}{n}}_{2,\;eff}$ was calculated using the following [21]:

(3) $${\overset{-}{n}}_{2,\;eff}=\;\Delta n/I$$

where Δ*n* is the refractive index change, and *I* is the light intensity in the MRR given by the following equation [21]:

(4) $$I\;=\frac{P_{\mathrm{in}}\times \mathrm{BUF}}{A_{eff\;}}$$

In Equation (4), *P_in_* is the input power, *A_eff_* is the effective mode area [20], and *BUF* is the intensity build-up factor of the MRR, which can be expressed as follows [45]:

(5) $$\mathrm{BUF}=\;\frac{(1\;-\;t_{1}^{2})t_{2}^{2}a^{2}}{1\;-\;2t_{1}t_{2}a+{\left(t_{1}t_{2}a\right)}^{2}}$$

where *t*_1,2_ and *a* are the fit MRR parameters in Table 1.

In Figure 3d, the average values of the extracted ${\overset{-}{n}}_{2,\;eff}$ for the TE and TM polarizations are ~3.861 × 10^−13^ cm^2^/W and ~3.357 × 10^−13^ cm^2/^W, respectively. The difference in these responses can be attributed to the asymmetric cross section of the high-index doped silica waveguide that results in different optical field distributions for the two modes. Based on the results in Figure 3d, we further extracted the ${\overset{-}{n}}_{2}$ for the doped silica material, denoted as ${\overset{-}{n}}_{2,\;\mathrm{doped}\;\mathrm{silica}}$, according to the following [20]:

(6) $${\overset{-}{n}}_{2,\;eff}=\frac{\iint_{D}^{}{n_{0}}^{2}\left(x,\;y\right){\overset{-}{n}}_{2}\left(x,\;y\right){S_{z}}^{2}\mathrm{dxdy}}{\iint_{D}^{}{n_{0}}^{2}\left(x,\;y\right){S_{z}}^{2}\mathrm{dxdy}}$$

where *D* is the integral of the optical fields over the material regions, *S_z_* is the time-averaged Poynting vector calculated using Lumerical FDTD commercial mode solving software, and *n*_0_ (*x*, *y*) and ${\overset{-}{n}}_{2}$ (*x*, *y*) are the linear refractive index and ${\overset{-}{n}}_{2}$ profiles over the waveguide cross section, respectively. The value of ${\overset{-}{n}}_{2}$ for silica used in our calculation was ~2.5 × 10^−13^ cm^2^/W [31,46]. Figure 3e shows the extracted ${\overset{-}{n}}_{2,\;\mathrm{doped}\;\mathrm{silica}}$ versus the input power. The average values of ${\overset{-}{n}}_{2,\;\mathrm{doped}\;\mathrm{silica}}$ derived from the TE and TM modes were ~3.7 × 10^−13^ cm^2^/W and ~3.1 × 10^−13^ cm^2^/W, respectively. The close resemblance between them reflects that the doped silica did not exhibit significant anisotropy in terms of its ${\overset{-}{n}}_{2}$. The results in Figure 3e also confirm that the predominant cause of the observed nonlinearity was thermal in nature. This is also supported by the fact that the Kerr nonlinear coefficient of doped silica (~1.3 × 10^−15^ cm^2^/W [47]) was over two orders of magnitude lower. Although there are minor fluctuations in ${\overset{-}{n}}_{2,\;\mathrm{doped}\;\mathrm{silica}}$ across various input powers in Figure 3d, these variations are not significant. Considering the limited input power range (i.e., ~2 mW to ~16 mW), it can be inferred that the ${\overset{-}{n}}_{2,\;\mathrm{doped}\;\mathrm{silica}}$ values will exhibit a relatively stable behavior [17]. Hence, the slight power-dependent variations in ${\overset{-}{n}}_{2,\;\mathrm{doped}\;\mathrm{silica}}$ are likely attributable to measurement errors.

## 5. Optical Bistability

**Figure 4:**
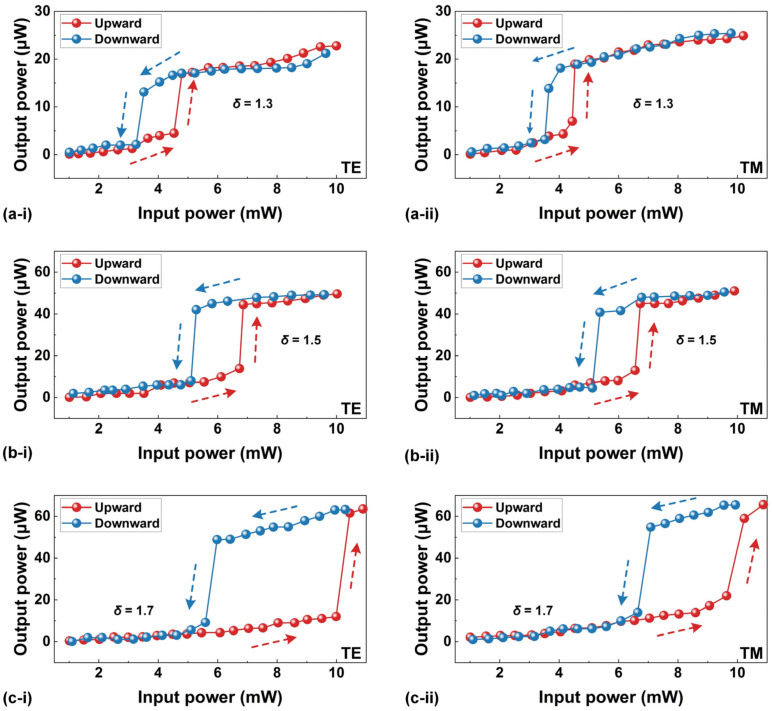
Measured output power versus input power with initial wavelength detunings of (**a**) *δ* = ~1.3, (**b**) *δ* = ~1.5, and (**c**) *δ* = ~1.7. In (**a**–**c**), (**i**) and (**ii**) show the results for TE- and TM-polarized resonances centered at ~1550.3758 nm and ~1550.2826 nm, respectively. Point-by-point measurements were taken at an average rate of ~1 Hz. The red and blue arrows indicate the increasing and decreasing of the input power, respectively.

Due to a steepened asymmetric transitional edge, optical bistability arising from nonlinear thermo-optic effects has been used to control light with light and achieve optical switches [9,10]. Figure 4 shows the measured output power as a function of the input power when it was progressively increased from ~1 mW to ~8 mW. For comparison, we also plotted the downward output power as the input power was subsequently reduced back to ~1 mW. In Figure 4a–c, we show the results for three initial wavelength detunings of *δ* = ~1.3, ~1.5, and ~1.7, respectively. The *δ* is defined as follows:

(7) $$\delta =(\lambda_{\mathrm{laser}}-\lambda_{\mathrm{res}})/\Delta \lambda$$

where *λ_laser_* is the wavelength of the input CW light, *λ_res_* is the resonance wavelength of the MRR measured at a low input CW power of ~0 dBm (i.e., the same as that in Figure 1 and does not induce significant asymmetry in the measured resonance spectral lineshape), and Δ*λ* is the 3 dB bandwidth of the resonance. In our measurements, we chose a TE-polarized resonance centered at *λ_res_* = ~1550.3758 nm and a TM-polarized resonance centered at *λ_res_* = ~1550.2826 nm. During the measurements, the maximum polarization extinction ratios were kept >30 dB.

In Figure 4a–c, redshifts of the resonance wavelengths can be observed for both TE and TM polarizations. During the upward sweeping, the output power first exhibited a steady and continuous increase, followed by a sudden jump toward higher output power. Conversely, during the downward sweeping with decreasing input power, there was a sudden jump toward lower output power after a gradual decrease in the output power. Clearly, the presence of a hysteresis loop resulting from the upward and downward wavelength sweeping provides evidence for the existence of optical bistability in the high-index doped silica MRR [48]. As *δ* was increased from ~1.3 to ~1.7, the input power threshold for optical bistability increased, and the hysteresis loop became more open. These phenomena are similar to those observed in Refs. [49,50]. We also note that the TE-polarized resonance exhibited a more open hysteresis loop compared with the TM-polarized resonance at the same *δ*. This observation shows agreement with the relatively large redshift of the resonance wavelength for the TE polarization in Figure 3b.

**Figure 5:**
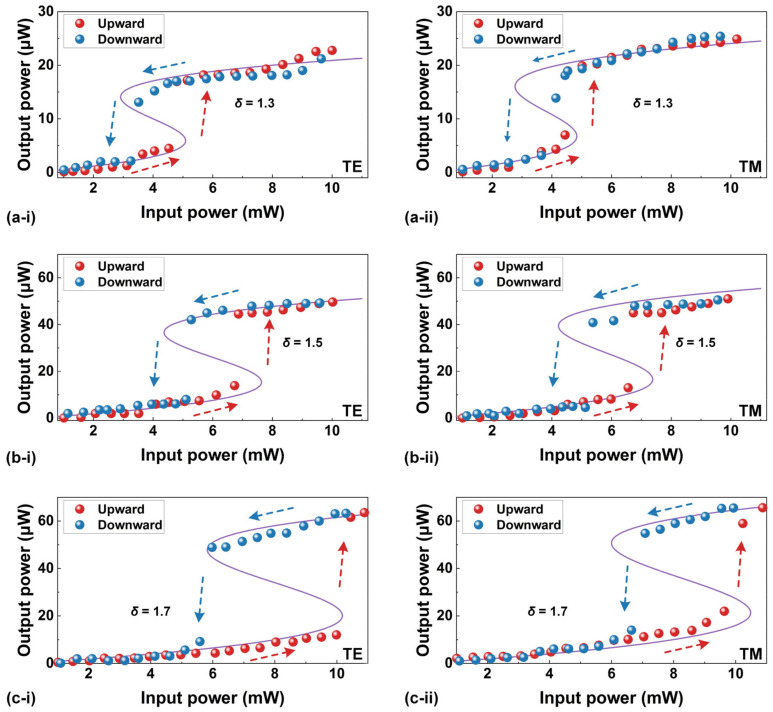
Measured (data points) and theoretical (solid curves) output power versus input power with initial wavelength detunings of (**a**) *δ* = ~1.3, (**b**) *δ* = ~1.5, and (**c**) *δ* = ~1.7. In (**a**–**c**), (**i**) and (**ii**) show the results for TE- and TM-polarized resonances. The red and blue arrows indicate the increasing and decreasing of the input power, respectively.

Figure 5a–c show the measured and theoretical output powers versus the input power for δ = ~1.3, ~1.5, and ~1.7, respectively. In each figure, we show the results for both TE and TM polarizations. The theoretical curves were calculated based on the theory in Refs. [29,48], using both the device parameters in Table 1 and the fit ${\overset{-}{n}}_{2,\;\mathrm{doped}\;\mathrm{silica}}$ in Figure 3e. In principle, bistable behavior occurs in the resonator response because, under specific conditions, the output power yields multiple distinct solutions for a given input power. Consequently, the resonator can switch between these solutions due to the influence of noise [29]. In Figure 5, the measured results show good agreement with the theoretical curves, providing further confirmation of the accuracy of the fit thermo-optic property parameters for the high-index doped silica devices.

## 6. Thermal Conductivity

The thermal conductivity, a parameter that defines a material’s ability to conduct heat, has been widely used to model thermal transport for applications related to thermal management, sensing, and energy storage [51,52,53,54,55]. In this section, the thermal conductivity of high-index doped silica is characterized by fitting the measured transmission spectra of the doped silica MRR at various input powers with theoretical simulations.

**Figure 6:**
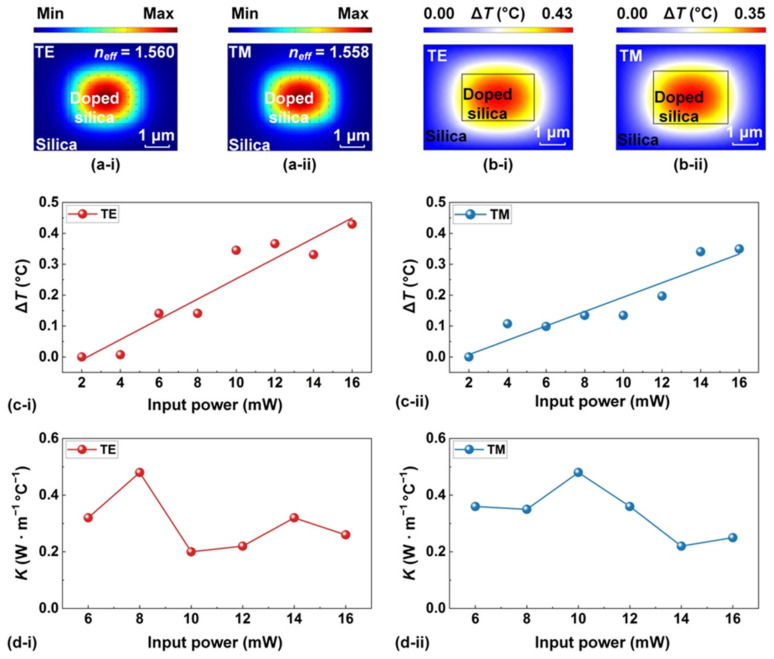
(**a**) Optical mode profiles of high-index doped silica waveguide for (**i**) TE and (**ii**) TM modes. (**b**) Temperature distribution profiles of high-index doped silica waveguide for (**i**) TE and (**ii**) TM modes. In (**a**,**b**), the input CW power is ~16 mW, and the initial temperature is assumed to be at the room temperature of 23 °C. (**c**) Calculated temperature variation versus input power for (**i**) TE and (**ii**) TM modes. (**d**) Thermal conductivity *K* versus input power for (**i**) TE and (**ii**) TM modes.

Figure 6(a-i,a-ii) show the simulated TE- and TM-mode profiles for the high-index doped silica waveguide. The corresponding effective refractive indices were *n_eff_TE_* = ~1.560 and *n_eff_TM_* = ~1.558 at 1550 nm. To further investigate the heat generated in the doped silica waveguide, we simulated the cross-sectional temperature distribution for both TE and TM polarizations. Figure 6(b-i,b-ii) show the steady-state temperature distributions at an incident power of 16 mW, which were obtained by solving the heat Equation [1]:

(8) −∇· (K∇T)=q

where *T* is the steady-state temperature distribution, *K* is the thermal conductivity, and *q* is the heat flux intensity. In Equation (8), ∇*T* denotes the gradient of *T*, and ∇ acting on the vector function *K*∇*T* is the corresponding divergence operator. In our simulation, the heat source power density *D* was calculated based on the TE- and TM-mode profiles in Figure 6(a-i,a-ii) using the following [56]:

(9) $$D=\;\frac{1}{2}\sigma {\left|E\right|}^{2}$$

where *σ* is the electrical conductivity of the waveguide in Table 1 and *E* is the amplitude of the optical field simulated in Figure 6a. It is worth noting that the build-up factor *BUF* in Equation (5) was taken into account when calculating the optical intensity in the MRR. In our simulation, the initial temperature *T*_0_ was set to 23 °C, which was the ambient temperature during the experiments.

When there are changes in the input power, the material conducts heat, leading to a rise in temperature and a redshift of the resonance wavelength. According to the results in Figure 2c and Figure 3c, we calculated the device temperature variation versus the input power. As shown in Figure 6c, at an input power of 16mW, the temperature variations for the TE and TM modes were Δ*T* = ~0.4298 °C and ~0.3495 °C, respectively. By fitting these temperature variations with the temperature distributions in Figure 6b, we obtained the thermal conductivity for the high-index doped silica, as shown in Figure 6d. For the TE and TM polarizations, the average values for the fitted thermal conductivity were ~0.30 W/(m·°C) and ~0.34 W/(m·°C). We note that the thermal conductivity of the high-index doped silica is lower than that of silica (i.e., ~1.4 W/(m·°C) [56]). This can be attributed to the introduction of the doping material, which slows down the lattice vibration coupling and the energy transfer. The specific values depend on the type and concentration of the doping element used, as well as the material’s fabrication method and structure. Based on Equation (8), the low thermal conductivity of the high-index doped silica waveguide restricts heat propagation, leading to a higher concentration of thermal energy within the waveguide. Consequently, this amplifies the temperature increase, which, in turn, facilitates the attainment of more pronounced optical bistability.

## 7. Comparison with Other Integrated Platform Materials

In this section, we present a summary of the thermo-optic property parameters of the doped silica devices obtained in Section 3, Section 4, Section 5 and Section 6, together with a comparison of them with those exhibited by other materials used for CMOS-compatible integrated photonic platforms. As shown in Table 2, the thermo-optic coefficient of high-index doped silica is higher than that of silica, but lower than those of silicon nitride and silicon. This can be attributed to the moderate refractive index of high-index doped silica among these materials. In terms of the coefficient characterizing the efficiency for the optically induced thermo-optic process, high-index doped silica exhibits a value that is below that of silicon, yet it surpasses those of silica and silicon nitride. This highlights its capability for implementing high-performance nonlinear thermo-optic devices. For the thermal conductivity, high-index doped silica displayed the lowest value among these materials. This benefits its applications for thermal mode locking in optical microcomb generation [7]. In the process of optical microcomb generation, the diminished thermal conductivity of high-index doped silica introduces a slow thermal reaction that influences the steady-state dynamics of the intracavity power. This, in turn, leads to a gradual correlation between the cavity detuning and the pump power. Such a characteristic decreases the rate of adjustment for power augmentation within the cavity in order to generate optical microcombs. As a result, it becomes feasible to achieve the simple generation of stable soliton crystal microcombs through manual tuning of the pump laser [19].

## 8. Conclusions

In summary, we provide detailed experimental characterization and theoretical analysis of the thermo-optic effects in integrated high-index doped silica devices that have been successfully applied in various linear and nonlinear optical applications. By fitting the experimental results with theory, we obtain fundamental parameters that define the thermo-optic performance of high-index doped silica devices, including the thermo-optic coefficient, the efficiency for the optically induced thermo-optic process, and the thermal conductivity. We also compare these parameters with those of other materials used for CMOS-compatible integrated photonic platforms, such as silicon, silicon nitride, and silica. Our finding provides valuable insights into the thermo-optic properties of high-index doped silica devices, which are crucial for effectively controlling and engineering these devices across diverse applications.

## Figures and Tables

**Table 1:** Device parameters of high-index doped silica MRR.

	Parameter	Symbol	Value	Source
Material parameters	Refractive index	*n*	silica: 1.45 doped silica: 1.60	[25]
	Electrical conductivity (S/m)	*σ*	6 × 10^−3^	[17]
Waveguide parameters	Width (μm)	*W*	3	Device structural parameter
	Height (μm)	*H*	2	Device structural parameter
MRR parameters	Ring radius (µm)	*R*	592.1	Device structural parameter
	Field transmission coefficients	*t*_1,2_ ^(a)^	TE: 0.9991 TM: 0.9992	Fit results from Figure 1d
	Round-trip amplitude transmission	*a*	TE: 0.9906 TM: 0.9875	Fit results from Figure 1d
	Intensity build-up factor	*BUF*	TE: 47.7 TM: 36.8	Calculated based on the fitted *t*_1,2_ and *a*

^(a)^ The field transmission coefficients of the two couplers formed by the MRR and the two bus waveguides are assumed to be equal, i.e., *t*_1_ = *t*_2_.

**Table 2:** Comparison of thermo-optic property parameters of high-index doped silica and other integrated platform materials.

Parameter	Thermo-Optic Coefficient (°C^−1^)	Coefficient for Optically Induced Thermo-Optic Process (cm^2^/W)	Thermal Conductivity (W·m^−1^ °C^−1^) ^(c)^	Refs.
Silicon	~1.8 × 10^−4^ (~86 pm/°C) ^(a)^	~7.8 × 10^−11^	~149	[33,57,58,59]
Silicon nitride	~2.6 × 10^−5^ (~11 pm/°C) ^(a)^	~1.5 × 10^−15^	~29	[21,60,61]
Silica	~1.1 × 10^−5^ (~15 pm/°C) ^(a)^	~2.5 × 10^−13^	~1.4	[31,33,46]
High-index doped silica ^(b)^	~1.46 × 10^−5^ (~13.8 pm/°C) ^(a)^	~3.4 × 10^−13^	~0.32	This work

^(a)^ Here, we also show the corresponding results for the wavelength shifts of resonators caused by temperature variation. Note that these results may vary based on the specific device used. ^(b)^ Here, we show the average values of the results for the TE and TM polarizations obtained in Section 3, Section 4, Section 5 and Section 6. ^(c)^ Note that the thermal conductivity may change with temperature, and here, we show the results at room temperature.

## Data Availability

Data are contained within the article.
